# Cytolethal Distending Toxin Enhances Radiosensitivity in Prostate Cancer Cells by Regulating Autophagy

**DOI:** 10.3389/fcimb.2017.00223

**Published:** 2017-06-08

**Authors:** Hwai-Jeng Lin, Hsin-Ho Liu, Chia-Der Lin, Min-Chuan Kao, Yu-An Chen, Chuan Chiang-Ni, Zhi-Pei Jiang, Mei-Zi Huang, Chun-Jung Lin, U-Ging Lo, Li-Chiung Lin, Cheng-Kuo Lai, Ho Lin, Jer-Tsong Hsieh, Cheng-Hsun Chiu, Chih-Ho Lai

**Affiliations:** ^1^Division of Gastroenterology and Hepatology, Department of Internal Medicine, School of Medicine, College of Medicine, Taipei Medical UniversityTaipei, Taiwan; ^2^Division of Gastroenterology and Hepatology, Department of Internal Medicine, Shuang-Ho HospitalNew Taipei, Taiwan; ^3^Division of Urology, Department of Surgery, Taichung Tzu Chi General HospitalTaichung, Taiwan; ^4^Department of Bio-Industrial Mechatronics Engineering, National Taiwan UniversityTaipei, Taiwan; ^5^Department of Otolaryngology-Head and Neck Surgery, China Medical University and HospitalTaichung, Taiwan; ^6^Department of Medical Research, School of Medicine, Graduate Institute of Basic Medical Sciences, China Medical University and HospitalTaichung, Taiwan; ^7^Department of Microbiology and Immunology, Graduate Institute of Biomedical Sciences, College of Medicine, Chang Gung UniversityTaoyuan, Taiwan; ^8^Department of Pediatrics, Molecular Infectious Disease Research Center, Chang Gung Children's Hospital and Chang Gung Memorial HospitalTaoyuan, Taiwan; ^9^Department of Urology, University of Texas Southwestern Medical CenterDallas, TX, United States; ^10^Department of Life Sciences, National Chung Hsing UniversityTaichung, Taiwan; ^11^Graduate Institute of Clinical Medical Sciences, College of Medicine, Chang Gung UniversityTaoyuan, Taiwan; ^12^Department of Nursing, Asia UniversityTaichung, Taiwan

**Keywords:** *Campylobacter jejuni*, cytolethal distending toxin, cell cycle, autophagy, radioresistance

## Abstract

Cytolethal distending toxin (CDT) produced by *Campylobacter jejuni* contains three subunits: CdtA, CdtB, and CdtC. Among these three toxin subunits, CdtB is the toxic moiety of CDT with DNase I activity, resulting in DNA double-strand breaks (DSB) and, consequently, cell cycle arrest at the G2/M stage and apoptosis. Radiation therapy is an effective modality for the treatment of localized prostate cancer (PCa). However, patients often develop radioresistance. Owing to its particular biochemical properties, we previously employed CdtB as a therapeutic agent for sensitizing radioresistant PCa cells to ionizing radiation (IR). In this study, we further demonstrated that CDT suppresses the IR-induced autophagy pathway in PCa cells by attenuating c-Myc expression and therefore sensitizes PCa cells to radiation. We further showed that CDT prevents the formation of autophagosomes via decreased high-mobility group box 1 (HMGB1) expression and the inhibition of acidic vesicular organelle (AVO) formation, which are associated with enhanced radiosensitivity in PCa cells. The results of this study reveal the detailed mechanism of CDT for the treatment of radioresistant PCa.

## Introduction

Cytolethal distending toxin (CDT) produced by *Campylobacter jejuni* is composed of three subunits: CdtA, CdtB, and CdtC. CdtA and CdtC interact with the cell membrane, enabling CdtB translocation across the membrane, followed by delivery into the nucleus (Lara-Tejero and Galan, 2001). After nuclear translocation, CdtB possesses DNase I activity, which causes DNA damage and in turn leads to cell-cycle arrest and apoptosis (Lara-Tejero and Galan, 2000). Notably, our recent study revealed that CDT can overcome the radioresistance of prostate cancer (PCa) cells by intervening in the repair of radiation-induced double-strand breaks (DSB) (Lai et al., 2014). However, the detailed mechanisms underlying the effects of CDT on radioresistance in PCa cells require further investigation.

The incidence and mortality of PCa have increased steadily worldwide during the past few decades (Sim and Cheng, 2005). Radiation therapy is an effective modality for treating localized PCa. However, PCa often becomes resistant to radiation after a prolonged period of radiotherapy. DOC-2/DAB2 interactive protein (DAB2IP) is frequently lost in high-grade PCa and has been recognized as a potent tumor suppressor in PCa progression. DAB2IP deficiency allows PCa cells to obtain proliferative, anti-apoptotic potential (Xie et al., 2009) and undergo epithelial–mesenchymal transition (Xie et al., 2010), leading to increased metastases and cancer cell stemness (Yun et al., 2015), in which cells are resistant to radiation-induced apoptosis (Kong et al., 2010). We recently demonstrated that CDT synergistically sensitizes the effects of radiation on DAB2IP-knockdown PCa cells but not in the normal DAB2IP expression cells (Lai et al., 2014). In addition, CDT enhances radiation-induced DAB2IP-knockdown cell death is mediated via the degradation of double-strand DNA, cell cycle arrest, and activation of the apoptotic pathway. However, the effects of CDT are not obviously shown in DAB2IP normal expression PCa cells. Therefore, it is important to investigate the detail mechanism how CDT sensitizes PCa cells to radiation, particularly those with the DAB2IP-deficient radioresistant phenotype.

Knocking-down DAB2IP induces autophagy after treatment with radiation (Yu et al., 2012). In addition, the inhibition of c-Myc impairs autophagosome formation (Toh et al., 2013). Treatment of DAB2IP-deficient PCa cells with *C. jejuni* CDT decreases the expression level of c-Myc, suggesting that CDT suppresses c-Myc, resulting in the inhibition of the autophagy pathway and induction of DSB (Lai et al., 2014). However, whether the regulation of autophagy by CDT enhances radiosensitivity in DAB2IP-deficient PCa cells remains to be investigated. In this study, we provide evidence that CDT inhibits c-Myc, resulting in impaired autophagy and rendering radioresistant PCa cells sensitive to radiation.

## Materials and methods

### Cell culture

LAPC4 PCa cells were cultured in Iscove's Modified Dulbecco's Medium (IMDM) (Gibco, Grand Island, NY) supplemented with 5% fetal bovine serum and incubated in a humidified atmosphere containing 5% CO_2_. The shRNA system (pGIPZ-lentiviral-shRNAmir from Open Biosystems, Huntsville, AL) was used to knockdown (KD) endogenous DAB2IP. The DAB2IP control (shVector) and knockdown (shDAB2IP) cells were selected by using puromycin. The efficiency of DAB2IP knockdown in LAPC4 cells was confirmed by using qRT-PCR and western blot analysis as described previously (Xie et al., 2009).

### Ionizing radiation

LAPC4-KD cells were irradiated at room temperature in ambient air using the Faxitron RX-650 irradiator (Faxitron X-ray, Wheeling, IL) at the indicated doses described in each experiment.

### Preparation of recombinant CDT proteins

Recombinant His-tagged CDT subunits were cloned by following the standard protocols as described previously (Lin et al., 2011). The expressed His-tagged CdtA, CdtB, and CdtC fusion proteins were purified by metal affinity chromatography (Clontech, Palo-Alto, CA) and assessed by SDS-PAGE. Each purified protein was subjected to ToxinEraser (GenScript, Piscataway, NJ) for removing of endotoxin (Lai et al., 2015).

### Immunoprecipitation

Cell lysates were prepared and subjected to immunoprecipitation at 4°C overnight, using 10 μg monoclonal anti-HMGB1 antibody (Abcam, Cambridge, MA) according to manufacturer's instructions (Invitrogen). Precipitates were then subjected to western blot assay (Lai et al., 2011).

### Western blot assay

LAPC4-KD cells treated with CDT (200 nM), IR (2 Gy), or CDT combined with IR for 24 h were harvested and cell lysate was prepared. The samples were then resolved by 6–12% SDS-PAGE and transferred onto polyvinylidene difluoride membranes (Millipore). Membranes were probed with primary antibodies: Bax, PARP, cleaved caspase 9 (purchased from Proteintech, Chicago, IL), Atg5, Atg12, mTOR, p62/SQSTM1 (purchased from GeneTex, Irvine, CA), Bak and β-actin (purchased from Santa Cruz, CA), or HMGB1 (purchased from Abcam, Cambridge, UK). The membranes were then incubated with horseradish peroxidase–conjugated secondary antibody (Millipore, Temecula, CA). The proteins of interest were detected using the ECL Western Blot Detection Reagents (GE Healthcare, Piscataway, NJ) and visualized using X-ray film (Kodak, Rochester, NY). The signal intensity of each protein was quantified with the Image J software (National Institute of Health, Bethesda, MD) as described previously (Lin et al., 2016).

### Cell cycle analysis

LAPC4-KD cells were treated with CDT (0–500 nM), IR (2 Gy), or CDT combined with IR. Cells were then incubated at 37°C for 0.5, 2, 8, 12, 24, and 48 h. The treated cells were harvested and fixed with ice-cold 70% ethanol for 1 h, and stained with 20 μg/ml propidium iodide (Sigma-Aldrich) containing 1 mg/ml RNase (Sigma-Aldrich) for 1 h. The stained cells were determined by an FACScalibur flow cytometer (Becton-Dickinson, San Jose, CA) and the data were analyzed using Cell Quest software WinMDI (Verity Software House, Topsham, Me) (Lai et al., 2013).

### Acridine orange staining

Cells (1 × 10^6^ cells/well) were seeded on 13-mm glass coverslip in 6-well plates. After treatment, cells were washed and stained with 1 μg/ml acridine orange (AO) for 15 min to visualize acidic vesicular organelles (AVO). AO-stained stained cells were observed under a fluorescence microscope (Carl Zeiss, Göttingen, Germany) and analyzed by using a flow cytometer (Becton-Dickinson). All samples were examined in three independent experiments.

### Immunofluorescence staining

After treatment, LAPC4-KD cells were washed and fixed with 1% paraformaldehyde (Sigma-Aldrich) followed by blocking with 1% BSA for 1 h. Cells were probed with LC3B antibody (Cell Signaling) at room temperature for 1 h and then incubated with Alexa Fluor 555-conjugated anti-rabbit antibody (Invitrogen) for 1 h (Xu et al., 2007). Nuclei were counterstained with Hoechst 33342 for 10 min. The stained cells were then analyzed using a fluorescence microscope (Carl Zeiss) as described previously (Liao et al., 2017).

### Statistical analysis

Statistical analyses for the data between two groups were determined using Student *t*-test. Statistics analysis comparisons of more than two groups were evaluated using two-way analysis of variance (ANOVA). *P* < 0.05 was considered statistically significant. The statistical software was the SPSS program (version 12.0 for windows, SPSS Inc., Chicago, IL).

## Results

### CDT suppresses autophagy in DAB2IP-deficient PCa cells

**Figure 1:**
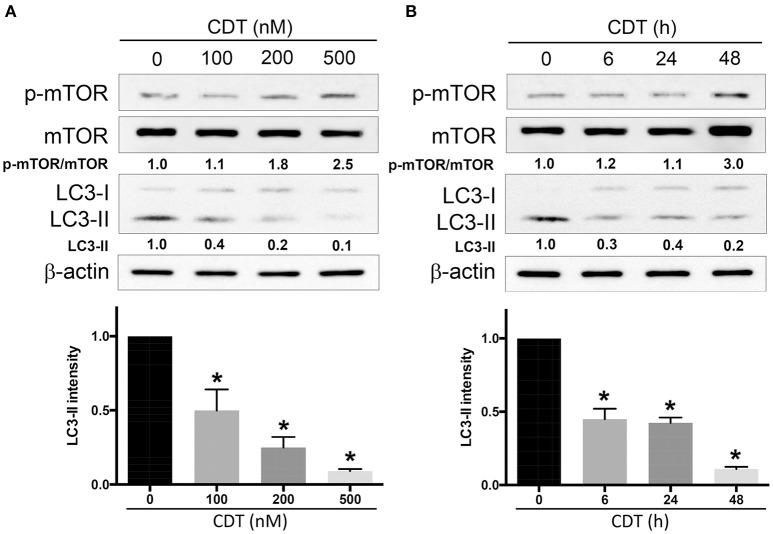
**CDT suppresses autophagy in DAB2IP-knockdown PCa cells**. LAPC4-KD cells were treated with **(A)** CDT (0–500 nM) for 24 h or **(B)** 200 nM CDT for 0–48 h. Cell lysates were subjected to western blot analyses using antibodies against phospho-mTOR, total mTOR, and the autophagy marker LC3-II. β-actin was used as a loading control. The protein expression levels of p-mTOR/mTOR and LC3-II were quantified and indicated at the bottom of lanes. The ratios of protein expression in untreated cells were set to 1. ^*^P < 0.01.

Autophagy is considered an important therapeutic target in radiation oncology (Yu et al., 2012). Microtubule-associated protein 1 light chain 3 (LC3-II) is commonly employed as a specific marker for monitoring autophagosome formation (Mizushima and Yoshimori, 2007). In this study, we first performed a western blot analysis to determine whether CDT regulates autophagy in radioresistant PCa cells. LAPC4-KD cells, DAB2IP-knockdown with radioresistant phenotype PCa cells, were used as an assay platform in this study (Kong et al., 2010). As shown in Figure 1A, the expression levels of phospho-mTOR were gradually increased upon treatment with CDT in a dose-dependent manner (0–500 nM). However, CDT suppressed autophagy, as indicated by the reduction in the LC3-II expression. Similar results were obtained in cells exposed to 200 nM CDT for 0–48 h; phospho-mTOR expression was elevated after treatment of cells with CDT for 48 h. In contrast, the LC3-II expression decreased as incubation time increased (Figure 1B). These results show that CDT decreases the amount of LC3-II, indicating that CDT suppresses autophagy in DAB2IP-deficient PCa cells.

### Combined treatment of CDT and IR enhances radiosensitivity in PCa cells

**Figure 2:**
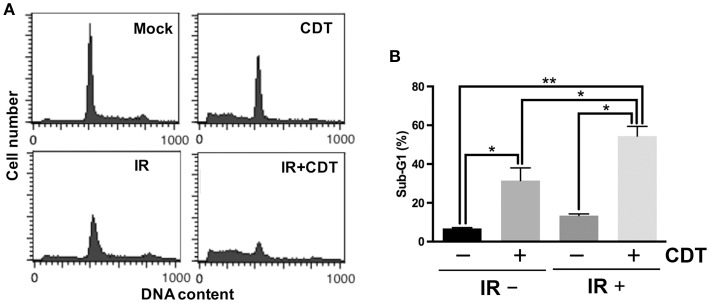
**CDT synergistically enhances IR-induced cell death in radioresistant PCa cells. (A)** LAPC4-KD cells were treated with CDT (200 nM), IR (2 Gy), or CDT combined with IR for 48 h. Cell cycle distribution was based on DNA content analyzed by flow cytometry. **(B)** The percentages of cells in sub-G1 phase were calculated and plotted. ^*^P < 0.05; ^**^P < 0.01.

We then analyzed whether CDT possesses activity to enhance radiosensitivity in PCa cells. Cells were treated with CDT alone, ionizing radiation (IR) alone, or IR combined with CDT, and the cell cycle distribution was assessed. As shown in Figure 2, only small proportions of sub-G1 cells were shown in untreated control and IR alone groups. However, an increased proportion of sub-G1 population was observed in cells treated with CDT or a combination of IR and CDT. At this point, we postulated that a combination treatment of CDT and IR increases sub-G1 population may be a consequence of the additive effect of CDT- and IR-induced cell death.

**Figure 3:**
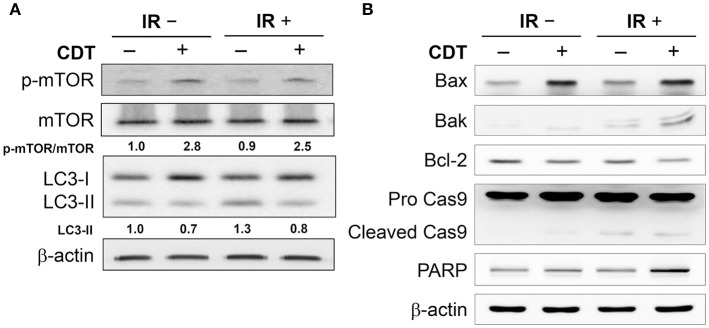
**CDT suppresses IR-induced autophagy in radioresistant PCa cells**. The protein expression levels of **(A)** p-mTOR, LC3-II, as well as **(B)** Bax, Bak, Bcl-2, cleaved caspase 9, and PARP in LAPC4-KD cells and those treated with CDT (200 nM), IR (2 Gy), and CDT combined with IR for 24 h are shown. β-actin was used as the loading control. The protein expression levels of p-mTOR/mTOR and LC3-II were quantified and indicated at the bottom of lanes. The ratios of protein expression in untreated cells were set to 1.

We next explored whether CDT manipulates autophagy in IR-treated PCa cells, the phosphorylation of mTOR and LC3-II expression in LAPC4-KD cells were analyzed. As shown in Figure 3A, phospho-mTOR expression was higher in cells treated with CDT or both CDT and IR than in those treated with IR alone. LC3-II, an autophagy specific marker, was also reduced in cells treated with either CDT alone or a combination of IR and CDT. Moreover, the expression levels of the apoptotic molecules Bax, Bak, PARP, and cleaved caspase 9 were significantly higher in cells treated with IR plus CDT than in those treated with IR alone (Figure 3B). In contrast, when cells were treated with IR and CDT, the expression of the anti-apoptotic molecule Bcl-2 decreased. These results demonstrate that CDT dampens autophagy in the conversion of radioresistant PCa cells to radiation-sensitive cells.

### CDT impairs autophagy in radioresistant PCa cells

**Figure 4:**
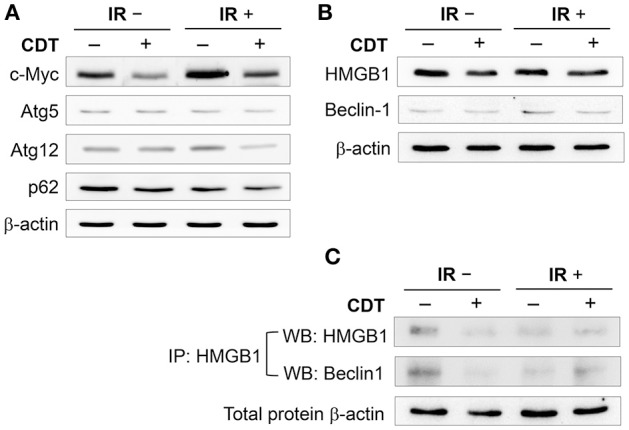
**CDT decreases autophagy induction**. LAPC4-KD cells were treated with CDT (200 nM), IR (2 Gy), or CDT combined with IR for 24 h. **(A)** The expression levels of c-Myc, Atg-5, Atg-12, and p62/SQSTM1 as well as **(B)** HMGB1 and Beclin1 in LAPC4-KD cells were assessed by western blot. **(C)** Cell lysates were subjected to immunoprecipitation (IP) and western blot assay for analyzing the expression of HMGB1 and Beclin1. β-actin was used as the loading control.

To further ascertain the role of CDT in the regulation of autophagy in IR-treated LAPC4-KD cells, we determined the expression levels of effector molecules involved in the autophagy pathway. Cells were treated or untreated with CDT (200 nM) and then irradiated at a dose rate of 2 Gy/min. The expression levels of molecules involved in the autophagy pathway, including c-Myc, Atg5, Atg12, and p62/SQSTM1, were analyzed by western blot. As shown in Figure 4A, treatment of cells with CDT combined with IR dramatically inhibited c-Myc expression when compared to the expression in the CDT-untreated or IR alone groups. In parallel, the expression levels of autophagy-related proteins, Atg5, Atg12, and p62/SQSTM1, were decreased in cells treated with a combination of CDT and IR compared to that in the CDT-untreated or IR alone groups. Binding of high-mobility group box 1 (HMGB1) to Beclin1 maintains Beclin1-phosphatidylinositol-3-kinase (PI3K) complex formation during autophagy activation (Tang et al., 2010); accordingly, we analyzed whether CDT reduces HMGB1, thereby limiting the formation of the HMGB1-Beclin1-PI3K complex and reducing autophagy. As shown in Figure 4B, the expression levels of Beclin1 and HMGB1 were reduced when cells were treated with IR combined with CDT. Furthermore, the IP experiment showed that a decrease of Beclin1 binding to HMGB1 upon treated with IR plus CDT (Figure 4C). In addition, CDT acts as a radiomimetic agent which induced persistent DNA damage (Fahrer et al., 2014) and delayed DSB repair (Lai et al., 2014). Taken together, these results reveal that CDT enhances radiosensitivity in PCa cells may be caused by induced DNA damage and impaired the autophagy process.

### CDT influences autophagy flux in radioresistant PCa cells

**Figure 5:**
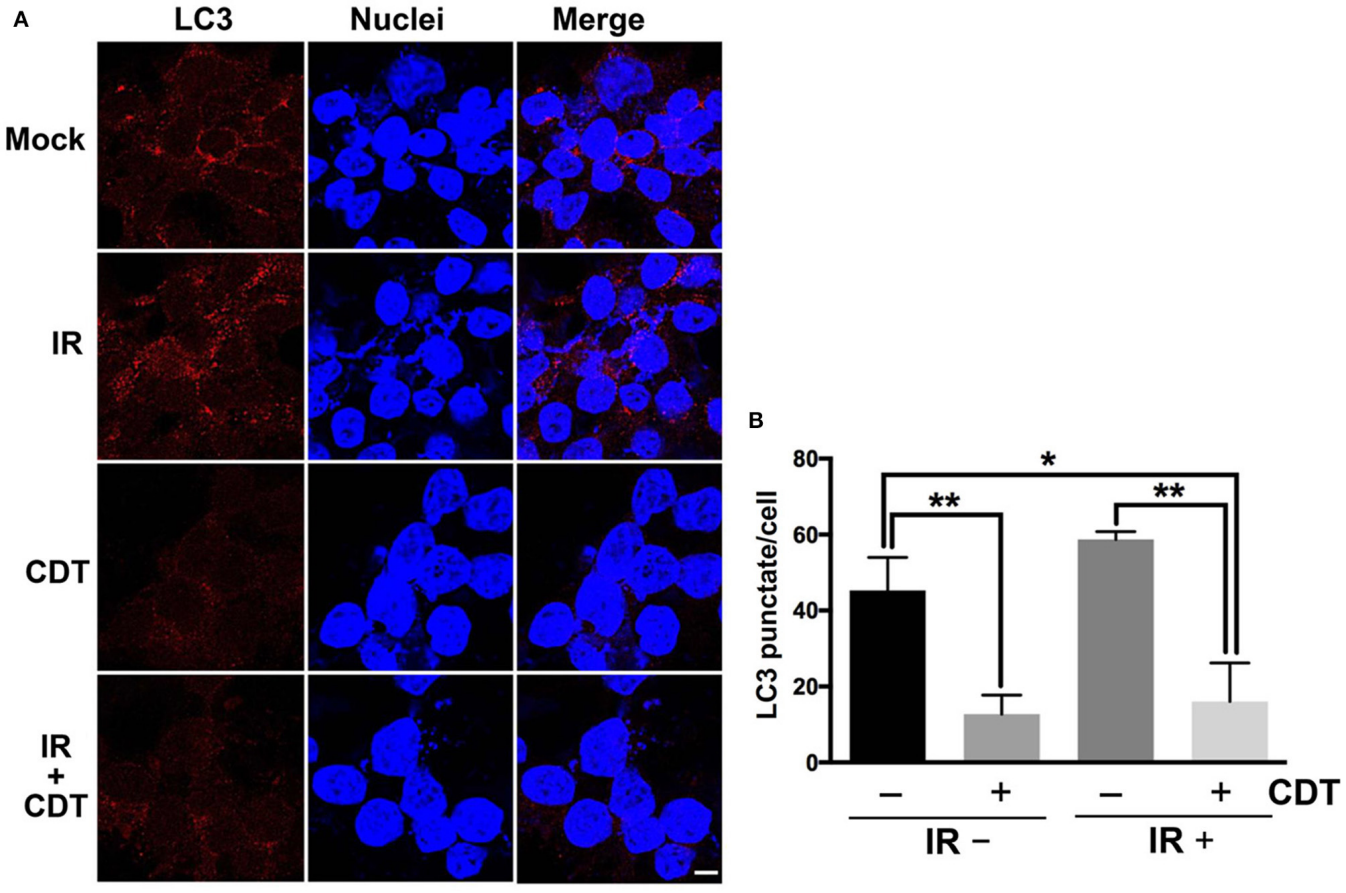
**CDT impairs autophagy in radioresistant PCa cells. (A)** LAPC4-KD cells were treated with CDT (200 nM), IR (2 Gy), or CDT combined with IR for 24 h. The treated cells were incubated for 24 h and stained with LC3-II (red) and Hoechst 33342 (blue) for visualization of autophagy and nuclei, respectively. Scale bar, 5 μm. **(B)** The number of LC3-II punctate formation were counted (50 cells were evaluated per sample). ^*^P < 0.05; ^**^P < 0.01.

**Figure 6:**
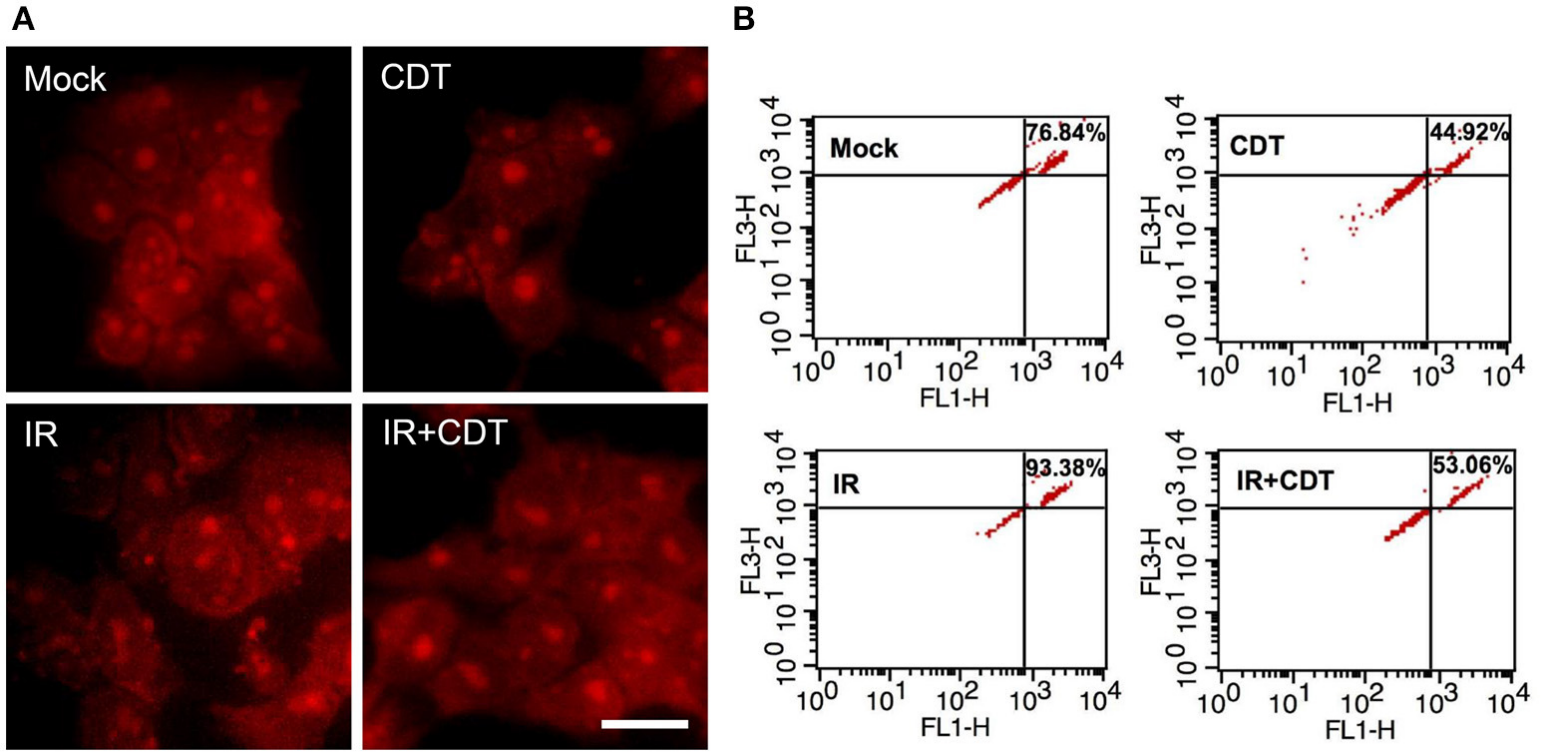
**CDT decreases IR-induced autophagosome formation**. LAPC4-KD cells were treated with CDT (200 nM), IR (2 Gy), or CDT combined with IR for 24 h. **(A)** The treated cells were stained with acridine orange (AO) to visualize the formation of acidic vesicular organelles (AVO). Scale bar, 20 μm. **(B)** Fluorescent AVO-containing cells were quantitatively evaluated by using flow cytometry analysis.

We then examined CDT-suppressed IR-induced autophagy using immunofluorescence staining for examining LC3 punctate formation (Xu et al., 2007). As shown in Figure 5, LC3 punctate formation was slightly increased in cells treated with IR compared to mock-treated cells. However, the punctate dots were significantly decreased in cells upon exposure to CDT alone or CDT combined with IR compared to that of IR-treated cells. During autophagy, the formation of AVO is increased and can be detected by staining with acridine orange (AO) (Yu et al., 2012). As shown in Figure 6, IR treatment caused an increase level of AO accumulation than that in the mock-treatment. We then observed that the amount of accumulated AO was decreased in cells treated with CDT alone or a combination of CDT and IR compared to IR-treated cells. Our data showed that AO staining and immunofluorescence autophagy detection were consistent with LC3-II expression, demonstrating that CDT suppresses IR-induced autophagy in response to radiosensitivity in PCa cells. Together, our results along with previous findings (Lai et al., 2014) unveil the potential mechanism by which CDT renders radioresistant PCa cells sensitive to radiation via the induction of DNA damage and suppression of the autophagy.

## Discussion

Bacterial toxins can be developed as treatment modalities for tumors (Forbes, 2010). The most beneficial properties of bacterial toxins are their easy purification and ability to specifically target the cell membrane followed by efficient delivery into cancer cells. Previous studies have examined several bacterial toxins in clinical settings for cancer therapy, including anthrax toxin (Liu et al., 2000), diphtheria toxin (Frankel et al., 2002), and Shiga toxin (Ishitoya et al., 2004). CDT can be produced from several gram-negative bacteria and its biochemical activity has been extensively characterized. *Aggregatibacter actinomycetemcomitans* CDT was first utilized for gingival squamous carcinoma therapy (Yamamoto et al., 2004). We recently demonstrated that *C. jejuni* CDT can be developed as a potential therapeutic agent for gastric cancer and radioresistant PCa (Lai et al., 2014, 2016). These studies provide support for the development of bacterial toxins as potential cancer therapeutic agents.

Interaction of CdtA and CdtC with membrane lipid rafts is required for CdtB internalization (Boesze-Battaglia et al., 2016). CdtB possesses DNase I activity, which leads to DSB (Lara-Tejero and Galan, 2000) and may regulate apoptotic or autophagy pathways (Robert et al., 2011). Autophagy is a lysosomal degradation pathway that eliminates damage or potentially dangerous cellular components under adverse conditions to protect organisms from metabolic stress (Kroemer et al., 2010). The regulation of autophagy is quite complicated and includes a variety of signaling mechanisms. Activation of HMGB1 can induce autophagy in cancer and immune cells (Zhang et al., 2013). In addition, a recent study in which HMGB1 was conditionally ablated in mice revealed that HMGB1 is required for the autophagy process (Yanai et al., 2013). Most importantly, the elevation of HMGB1 activates the autophagy pathway and has been implicated in the development of radioresistance in bladder cancer (Shrivastava et al., 2016). Loss of HMGB1 increases DNA damage and sensitizes cancer cells to radiation (Shrivastava et al., 2016). In addition, CDT possesses a genotoxic activity that has been shown to function as a radimimetic agent and prolong persistent levels of DNA damage (Fahrer et al., 2014). Together with the previous findings (Fahrer et al., 2014; Lai et al., 2014) and our current analyses showed that CDT reduces autophagy-related molecules, including LC3-II, Beclin1, and HMGB1, indicating that CDT renders radioresistant PCa cells sensitive to radiation may be attributed to the induction of DSB and suppression of the autophagy pathway.

**Figure 7:**
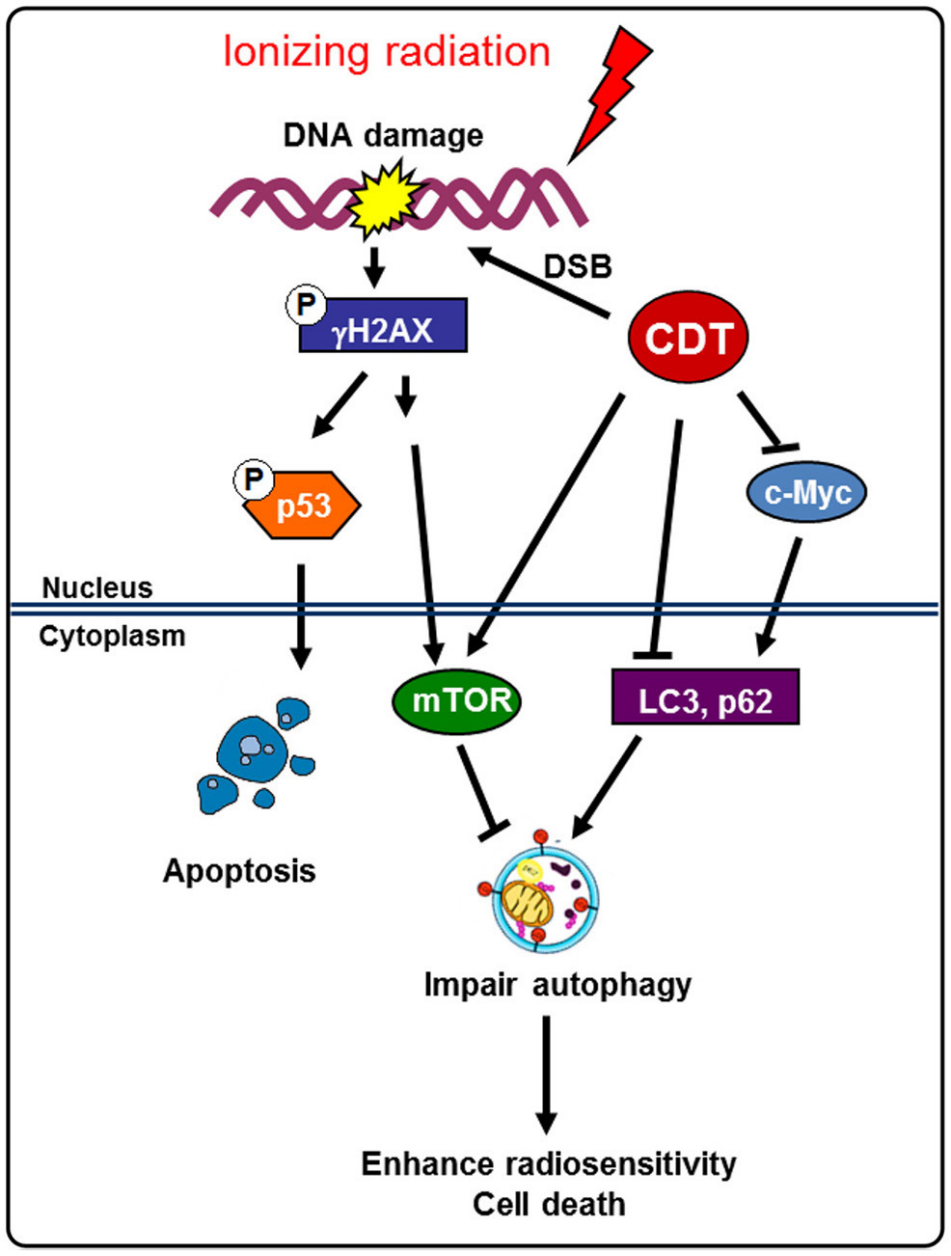
**Model for CDT enhances radiosensitivity in PCa cells**. Radiation-induced autophagy enhances radioresistance and leads to the activation of the survival pathway in DAB2IP-deficient PCa cells. A bacterial genotoxin CDT inhibits c-Myc and increase mTOR to impair autophagy induction, thereby rendering radioresistant PCa cells sensitive to radiation. This study elucidates the mechanisms by which CDT sensitizes radioresistance in PCa cells and provides a basis for its development as a target agent for the treatment of refractory PCa.

Cancer cells utilize autophagy as an adaptive and context-dependent system to overcome radiotherapeutic stress (Bergmann, 2007; Apel et al., 2008). In addition, in response to radiation and DNA damage or radioresistance, tumor cells may be influenced by autophagy regulation (Chaachouay et al., 2011). c-Myc promotes the growth, differentiation, apoptosis, and metabolism of cancer cells (Grandori et al., 2000). Additionally, DAB2IP inhibits the expression of c-Myc and suppresses the growth of PCa cells (Yu et al., 2012). Our recent study demonstrated that CDT can stimulate the expression of phosphoproteins, including γ-H2AX, ATM, and CHK2, which respond to DSB. We also showed that the treatment of DAB2IP-deficient cells with CDT decreases the expression of c-Myc. Since c-Myc is able to promote autophagosome formation (Toh et al., 2013), we further demonstrated that CDT suppresses the autophagy pathway and induction of DSB via the inhibition of c-Myc (Figure 7).

In conclusion, this study revealed that CDT is able to enhance the effects of radiotherapy in radioresistant PCa cells. Our previous studies and current findings demonstrate that CDT inhibits c-Myc and reduces HMGB1, resulting in prolonged IR-induced DSB and impaired autophagy, thus converting radioresistance to radiosensitivity in PCa cells. Understanding the importance of CDT activity and the molecular basis of the functions of the particular toxin will provide a novel strategy for eradicating radioresistant PCa.

## Author contributions

Conception or design of this work: J-TH, HL, C-HC, and C-HL. Experimental study: H-JL, H-HL, C-DL, M-CK, Y-AC, CC-N, Z-PJ, M-ZH, U-GL, L-CL, C-JL, and C-KL. Data analysis and interpretation: H-JL, H-HL, C-DL, C-HC, and C-HL. Writing the manuscript: J-TH, HL, C-HC, and C-HL. Final approval: all authors.

### Conflict of interest statement

The authors declare that the research was conducted in the absence of any commercial or financial relationships that could be construed as a potential conflict of interest. The reviewer JR and handling Editor declared their shared affiliation, and the handling Editor states that the process nevertheless met the standards of a fair and objective review.
